# Hemozoin impairs cell cycle progression and promotes chemokine release in human microvascular endothelial cells

**DOI:** 10.1186/1475-2875-11-S1-P9

**Published:** 2012-10-15

**Authors:** Nicoletta Basilico, Daniela Girelli, Sarah D’Alessandro, Yolanda Corbett, Mauro Prato, Silvia Carluccio, Serena Del Bue, Pasquale Ferrante, Donatella Taramelli

**Affiliations:** 1 Dipartimento di Scienze Biomediche, Chirurgiche e Odontoiatriche, Università di Milano, Milan, Italy; 2 Dipartimento di Scienze Farmacologiche e Biomolecolari, Universitá di Milano, Milan, Italy; 3 Dipartimento di Neuroscienze e Dipartimento di Genetica, Biologia e Biochimica, Universitá di Torino, Turin, Italy

## Background

Cerebral malaria (CM) is a fatal complication of *P.falciparum* infection caused by the cytoadherence of infected erythrocytes to brain endothelial cells followed by micro-circulatory obstruction, blood-brain barrier (BBB) damage, ring hemorrhages, inflammatory response and neurological sequelae. The combination of both parasite and host factors are involved in the pathogenesis of CM. In particular, malarial pigment, hemozoin (HZ) was shown to interfere with monocytes and endothelial cell functions. Recently our group demonstrated that HZ enhanced total gelatinolytic activity in endothelial cells by inducing ex novo matrix metalloproteinases-9 (MMP-9) and promoting proMMP-9 protein expression (Prato et al., 2011).

## Materials and methods

In the present work human dermal microvascular endothelial cells (HMEC-1) were treated with native HZ isolated from *P. flaciparum* cultures. Cell morphology was evaluated by optical microscopy, chemokine (CXCL-8, CCL-5) production by ELISA, proliferation/viability by MTT assay and trypan blue count, apoptosis and cell cycle by FACS analysis, using Annexin V*l* Propidium Iodide (PI) and only PI, respectively.

## Results

Modifications of cell morphology were observed in HZ-treated cells, which showed elongated form instead of the classical polygonal shape. Moreover, HZ stimulated the production of the chemokines CXCL-8 and CCL-5, involved in neutrophils/monocytes recruitment, in a dose and time-dependent manner. After 48-72 hours of culture in the presence of HZ, a reduction of cells number/proliferation, compared to the control, was observed. However, FACS analysis indicated that HZ treatment did not induce apoptosis of endothelial cells. On the contrary, HZ increased the percentage of GO/G1-phase cells and decreased S-phase cells, indicating a reduction of the growth fraction cells.

## Conclusions

The present data suggest that HZ promotes chemokines release and inhibits endothelial cells growth. As a consequence, neutrophils/monocytes recruitment is favoured resulting in the amplification of the inflammatory response. Moreover, in case of vascular injury, the damaged monolayer could not be regenerated by proliferating endothelial cells. All these events may concur to impair BBB functions in CM.

